# Supplementary figures and images for: Forecasting under-five stunting in Ethiopia using classical and machine learning time series models
Source: PLoS One. 2026 Apr 22;21(4):e0345000. doi: 10.1371/journal.pone.0345000 (PMC13102227; doi:10.1371/journal.pone.0345000)

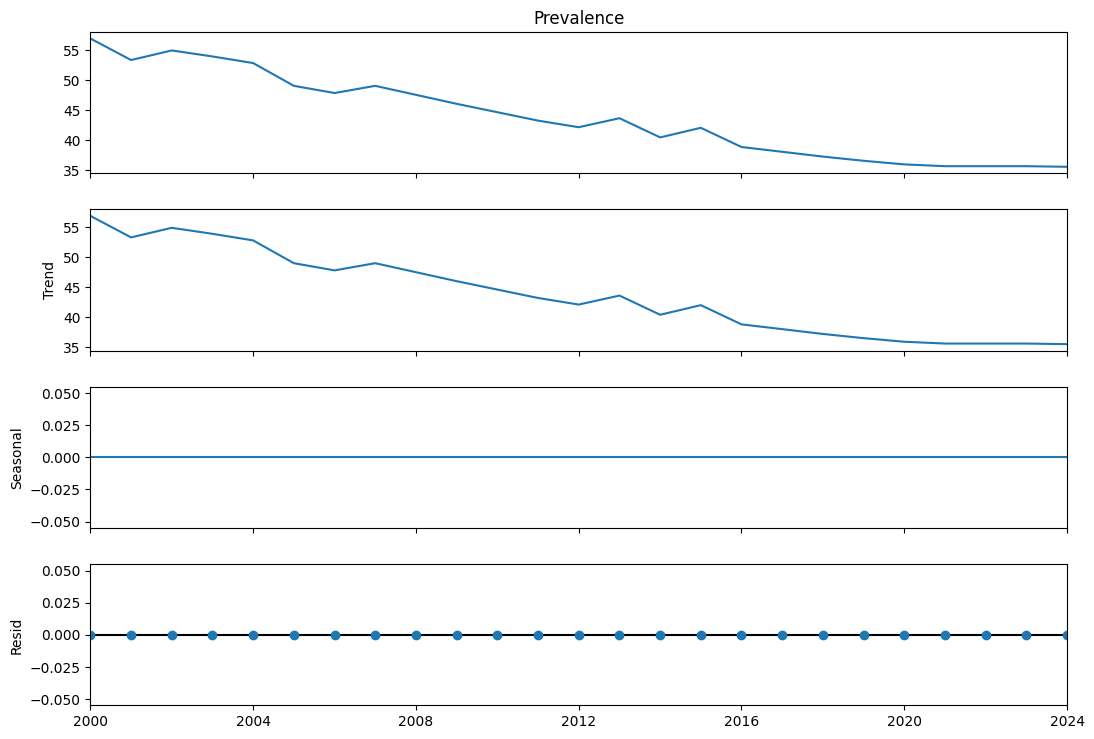

Supplement: S1 Fig — (TIF) [file pone.0345000.s001.tif]

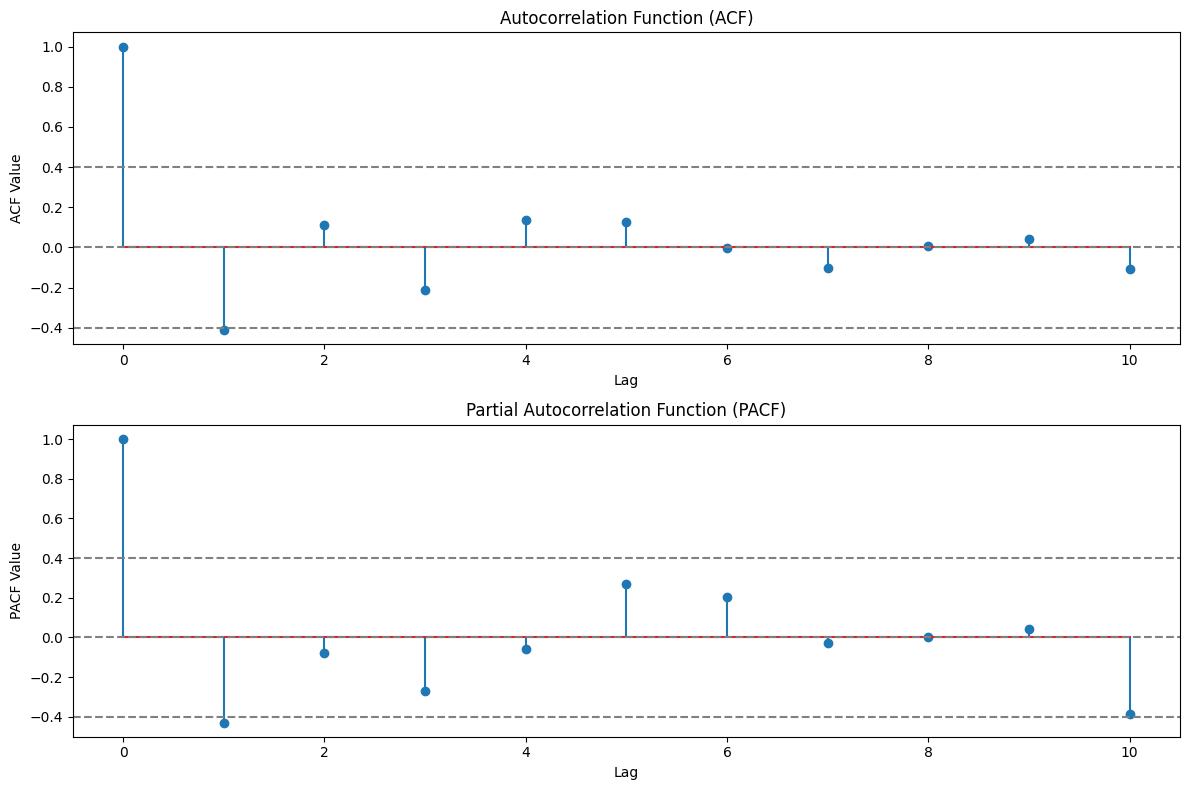

Supplement: S2 Fig — (STIF) [file pone.0345000.s002.tif]

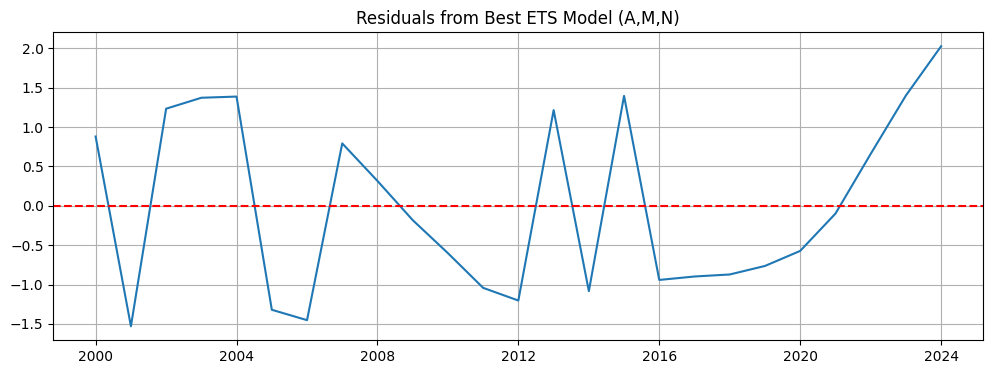

Supplement: S3 Fig — (TIF) [file pone.0345000.s003.tif]
