# Supplementary material for: Forecasting under-five stunting in Ethiopia using classical and machine learning time series models
Source: PLoS One. 2026 Apr 22;21(4):e0345000. doi: 10.1371/journal.pone.0345000 (PMC13102227; doi:10.1371/journal.pone.0345000)
Supplement: Attachment — Submitted filename: Response to Reviewers.docx [file pone.0345000.s006.docx]

Dear Editor and Reviewers,

Thank you very much for your valuable and constructive comments. We have carefully addressed all points, and the revisions have been incorporated as detailed in the table below. If there are any additional comments or suggestions, we would be very glad to address them.

We sincerely appreciate your time and support in improving our manuscript.

| **Comment / Requirement** | **Response / Revision** |
| --- | --- |
| **Reviewer #1:** The manuscript would benefit from clearer reporting of time series stationarity diagnostics. | We have added a detailed description of the stationarity assessment in the Methods section. Visual inspection of rolling statistics (mean and standard deviation) and formal Augmented Dickey-Fuller (ADF) tests are now reported. The original series was non-stationary (ADF p = 0.62), and stationarity was achieved after first differencing (ADF p < 0.001). Figures illustrating these checks are included as Figure 2 and 3. |
| **Reviewer #1:** A more detailed description of hyperparameter tuning for the machine learning models is needed. | The Methods section has been expanded. For MLP, hyperparameters (hidden layers, activation functions, learning rates, batch sizes, L2 regularization, max iterations) were tuned via randomized search with early stopping. For LSTM, hyperparameters (units, dropout rate, learning rate, batch size, number of epochs) were tuned via manual random search with early stopping and learning rate reduction callbacks. The final selected models and rationale for shallow architectures are explicitly described. |
| **Reviewer #1:** Exclusive reliance on annual data and absence of explanatory socioeconomic/contextual variables limit analytical depth. | We acknowledge this limitation. The Discussion now explicitly notes that forecasts are based on national-level annual data without socioeconomic or contextual covariates, which may limit the ability to account for drivers of stunting trends. Additionally, national-level data may obscure important regional or sub-population disparities. |
| **Reviewer #1:** The superior performance of the ETS model compared to machine learning approaches warrants further methodological discussion. | We added a detailed explanation of why ETS outperformed the machine learning models: 1) Small sample size increases overfitting risk for complex models, whereas ETS’s parsimonious structure provides stable forecasts. 2) ETS is well-suited for univariate forecasting and does not require covariates. 3) Smooth trends in the annual stunting series favor classical exponential smoothing methods. Relevant references have been included. |
| **Reviewer #1:** Conclusions could be strengthened by more specific, results-driven policy recommendations. | We revised the Conclusion section to include **results-driven recommendations** based on our projections: the projected under-five stunting prevalence (~32% by 2030) exceeds Ethiopia’s national target (19%) and the SDG 2.2 goal. We recommend focused research to identify factors driving high stunting prevalence and evidence-based, context-specific interventions to support achievement of national and global targets. |
| **Reviewer #2:** Well-organized paper; motivation is good; methods and analysis are thorough; minor editing issues (spacing, punctuation, etc.). | We thank the reviewer for the positive feedback. We have conducted an additional thorough proof-read of the manuscript to correct spacing, punctuation, and other typographical errors. |
| **Editor:** Ensure manuscript meets PLOS ONE style requirements, including file naming. | The manuscript has been reformatted according to the PLOS ONE style templates for both main body and title/authors/affiliations. All file names now comply with the journal guidelines. |
| **Editor:** Provide repository name and/or DOI/accession number or direct link in Data Availability Statement. | The Data Availability Statement has been updated to include the WHO Global Health Observatory repository and the direct link for the under-five stunting prevalence data: https://data.who.int/indicators/i/5F8A486?utm_source |
| **Editor:** Include captions for Supporting Information files and update in-text citations. | Captions for all Supporting Information files have been added at the end of the manuscript, and all in-text citations have been updated accordingly. |
| **Editor:** Review reviewer-recommended citations for relevance. | No additional citations were recommended by the reviewers. |
| **Editor:** Review and ensure references are complete and correct; remove or indicate retracted articles. | The reference list has been thoroughly reviewed. Any retracted articles have been removed or replaced, and all references are complete and formatted according to PLOS ONE guidelines. |
